# Discovering Dysfunction of Multiple MicroRNAs Cooperation in Disease by a Conserved MicroRNA Co-Expression Network

**DOI:** 10.1371/journal.pone.0032201

**Published:** 2012-02-22

**Authors:** Yun Xiao, Chaohan Xu, Jinxia Guan, Yanyan Ping, Huihui Fan, Yiqun Li, Hongying Zhao, Xia Li

**Affiliations:** College of Bioinformatics Science and Technology, Harbin Medical University, Harbin, Heilongjiang, China; University of North Carolina at Charlotte, United States of America

## Abstract

MicroRNAs, a new class of key regulators of gene expression, have been shown to be involved in diverse biological processes and linked to many human diseases. To elucidate miRNA function from a global perspective, we constructed a conserved miRNA co-expression network by integrating multiple human and mouse miRNA expression data. We found that these conserved co-expressed miRNA pairs tend to reside in close genomic proximity, belong to common families, share common transcription factors, and regulate common biological processes by targeting common components of those processes based on miRNA targets and miRNA knockout/transfection expression data, suggesting their strong functional associations. We also identified several co-expressed miRNA sub-networks. Our analysis reveals that many miRNAs in the same sub-network are associated with the same diseases. By mapping known disease miRNAs to the network, we identified three cancer-related miRNA sub-networks. Functional analyses based on targets and miRNA knockout/transfection data consistently show that these sub-networks are significantly involved in cancer-related biological processes, such as apoptosis and cell cycle. Our results imply that multiple co-expressed miRNAs can cooperatively regulate a given biological process by targeting common components of that process, and the pathogenesis of disease may be associated with the abnormality of multiple functionally cooperative miRNAs rather than individual miRNAs. In addition, many of these co-expression relationships provide strong evidence for the involvement of new miRNAs in important biological processes, such as apoptosis, differentiation and cell cycle, indicating their potential disease links.

## Introduction

MicroRNAs (miRNAs) are a class of small non-coding RNA molecules that ‘fine-tune’ gene expression on the posttranscriptional level. MiRNAs can negatively regulate their target genes by imperfect base pairing to the 3′-untranslated region (UTR) of their targets, which induce translational inhibition or deadenylation and mRNA decay. A large number of studies have demonstrated that miRNAs play important roles in a wide range of biological processes, such as development, differentiation and apoptosis. Furthermore, emerging evidence also indicates that miRNAs are involved in the pathogenesis of many human diseases, such as cancer and cardiovascular disease. Especially in cancer, miRNAs can function as oncogenes or tumor suppressors. Nonetheless, understanding of miRNA function and their roles in disease is still in its infancy.

A systematic genetic mutation study discovered that the majority of miRNA gene mutations in *Caenorhabditis elegans* do not result in obviously abnormal phenotypes [1]. A recent study further revealed that only few abnormal phenotypes are observed in *Caenorhabditis elegans* strains that each lack of multiple or all miRNA family members [2]. These results show that miRNAs may function together with other miRNAs. Many recent studies also found that some miRNAs can cooperatively control a variety of biological processes, such as cell development [3] and differentiation [4], [5], apoptosis [6], cell cycle [7], [8], and epithelial cell polarity [9]. Moreover, the multiplicity of miRNA targets can confer miRNAs the ability to cooperatively regulate a single biological process by targeting common components of that process. Using predicted targets, several bioinformatics studies have discovered many miRNA-mRNA modules [10], [11], [12], [13], [14], [15]. Our recent work also demonstrated potential functional relationships between miRNAs based on common targets [16]. Thus, it is reasonable to assume that miRNAs can function in a cooperative manner, rather than in a separate way. Exploring functional relationships between miRNAs may provide important clues about their function and how miRNAs contribute to human disease.

Over the last decade, microarrays have emerged as a powerful tool for comprehensively analyzing the expression levels for thousands of genes, and many studies utilized gene expression profiles to learn about gene functions [17], [18], [19], [20]. Like genes, miRNA microarrays have been widely used for exploring the roles of different miRNAs in various pathophysiological states. Many miRNA microarray studies have demonstrated that miRNAs can be used for disease diagnosis, prognosis and treatment [21], [22]. These large number of available miRNA expression profiles have been used to predict miRNA targets and analyze functional relationships between miRNAs. For example, Ritchie et al. [23] combined expression data from human and mouse to predict putative miRNA targets. A recent study completed by Volinia et al. [24] constructed miRNA networks in normal tissues and cancer using miRNA expression, and identified important miRNA cliques in cancer.

In this study, we performed a large-scale bioinformatics analysis of conserved miRNA co-expression relationships to systematically investigate functional links between miRNAs. By integrating human and mouse miRNA expression data, a conserved miRNA co-expression network was built. We confirmed that these conserved co-expressed miRNA pairs in the network are more likely to be functionally relevant. By mapping known disease miRNAs to the network, we identified three miRNA sub-networks that are highly related to cancer, and further explored their functions based on predicted targets and miRNA knockout/transfection expression data. Our results suggest that the pathogenesis of human disease may be associated with the impairment of functional cooperation between miRNAs.

## Results

### Construction of a conserved miRNA co-expression network

**Figure 1:**
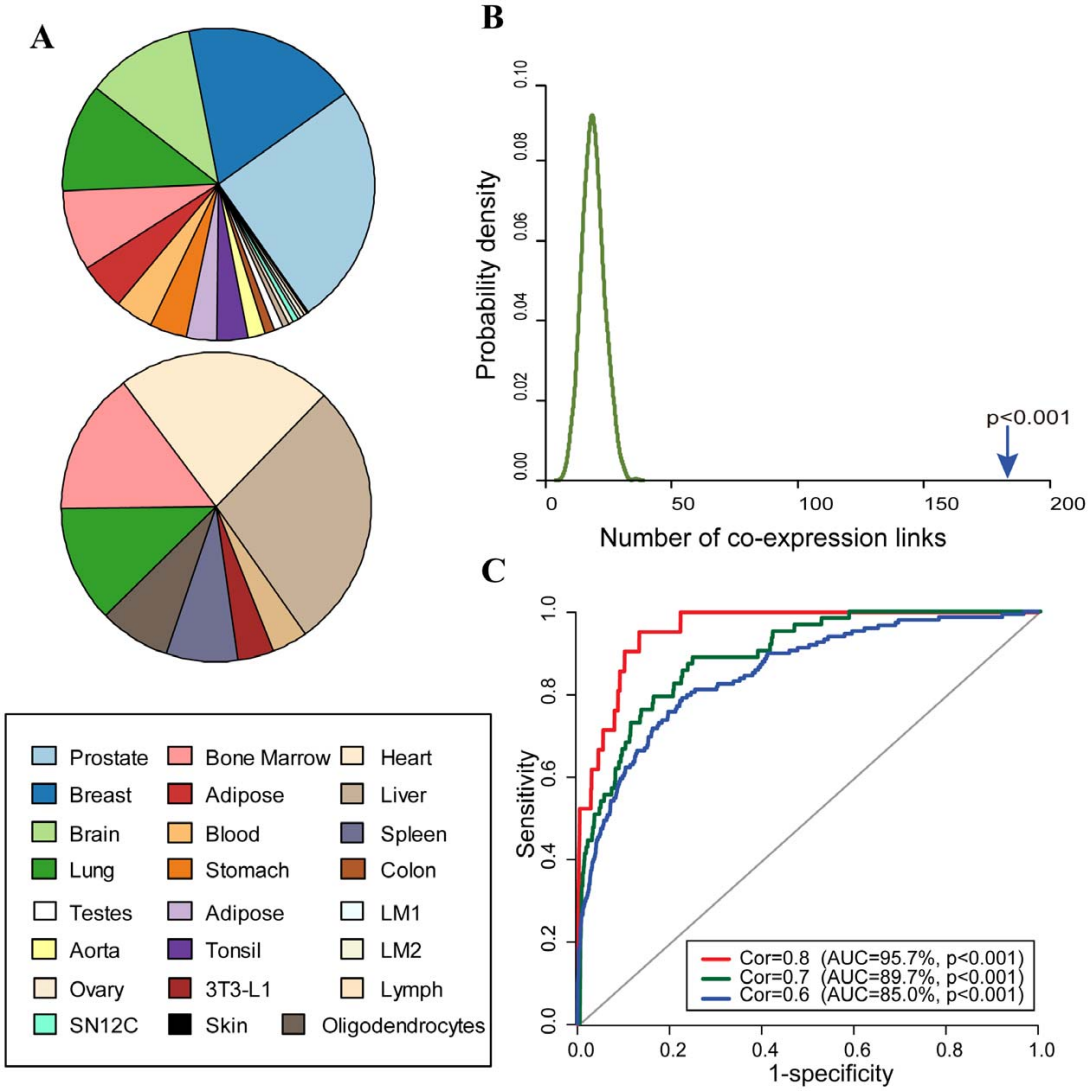
Evaluation of the conserved co-expression relationships. (A) Pie charts of miRNA expression data from human (top) and mouse (bottom) included in the analysis. Colors represent different tissues. (B) Probability density of the number of co-expression links identified through the permutation of orthologous miRNAs. The permutation experiment was repeated 100 times. (C) ROC curves used to quantify the significant of these relationships using a PCR-based miRNA expression data (GSE23024).

We collected 16 human and 8 mouse miRNA expression data sets respectively including 611 and 107 samples (Figure 1A). All expression data sets were generated using Agilent arrays. After normalization and probes mapping, 702 and 490 mature miRNAs were consistently present in human and mouse miRNA expression data sets, respectively. To identify miRNAs that are co-expressed across human and mouse, we identified 285 human-mouse orthologous miRNAs by all-against-all alignment of precursor miRNA (pre-miRNA) sequences with 11 bp flanking regions. Because all expression data sets used in this study are specific for mature miRNAs, we then linked mature miRNAs in human with their corresponding mature miRNAs in mouse according to these 285 orthologous miRNAs. Finally, 341 human-mouse orthologous mature miRNAs were identified. Of these, 253 with both members having expression measurements were used in the following analysis (Table S1).

Using the method described in [20], we identified 182 significant conserved co-expression relationships (corrected *p-value*<0.05). Based on these relationships, a conserved miRNA co-expression network with 163 nodes and 182 edges was constructed. In order to verify the significance of these conserved co-expressed relationships, we randomly permuted the orthologous mature miRNAs (i.e. random selections of miRNAs from human and mouse as putative human-mouse orthologous mature miRNAs), and constructed a conserved miRNA co-expression network using the permuted orthologous mature miRNAs. By repeating the permutation procedure 1000 times, we found significantly more co-expression relationships in the real conserved co-expression network than in the random networks (*p-value*<0.001, Figure 1B).

Because samples in the human and mouse miRNA expression data sets are derived from different tissues with different phenotypes, these conserved co-expression relationships may be influenced by sample heterogeneity. To determine whether these co-expression relationships are robust to the choice of samples, we randomly selected 80% of samples from human and mouse expression data sets for identifying conserved co-expression relationships. We repeated the procedure 1000 times. An average of 184 relationships was identified. Furthermore, we found that an average of 89.1% of relationships is also included in the network constructed using all samples. These results suggest that the majority of co-expression relationships are widespread in a variety of tissues and disease states, representing a general set of co-expression relationships.

To further evaluate the conserved co-expression relationships, we sought to use the receiving operator characteristic (ROC) curve, which provide a way of measuring sensitivity and specificity, to quantify the significant of these relationships. The area under the ROC curve (AUC) was used as a measure for the overall accuracy. Due to the absence of validated co-expression relationships, we constructed three sets of co-expression relationships according to different Pearson correlation thresholds using a PCR-based miRNA expression data (GSE23024). Our results illustrated in Figure 1C show that the conserved co-expression relationships achieve high AUC scores (>85.0%), which are significantly higher than random (*p-value*<0.001).

### Functional relationships between conserved co-expressed miRNA pairs

In order to further understand these conserved co-expression relationships, we retrieved the human network from the human-mouse conserved miRNA co-expression network by simply extracting human miRNAs in each node. We sought to analyze some characteristics of conserved co-expressed miRNA pairs in the human network. To determine the significance of these characteristics, we generated 1000 random sets with the same number of co-expression relationships as in the real network. For each randomization, we generated 182 miRNA pairs by randomly choosing two miRNAs and linking them together.

**Figure 2:**
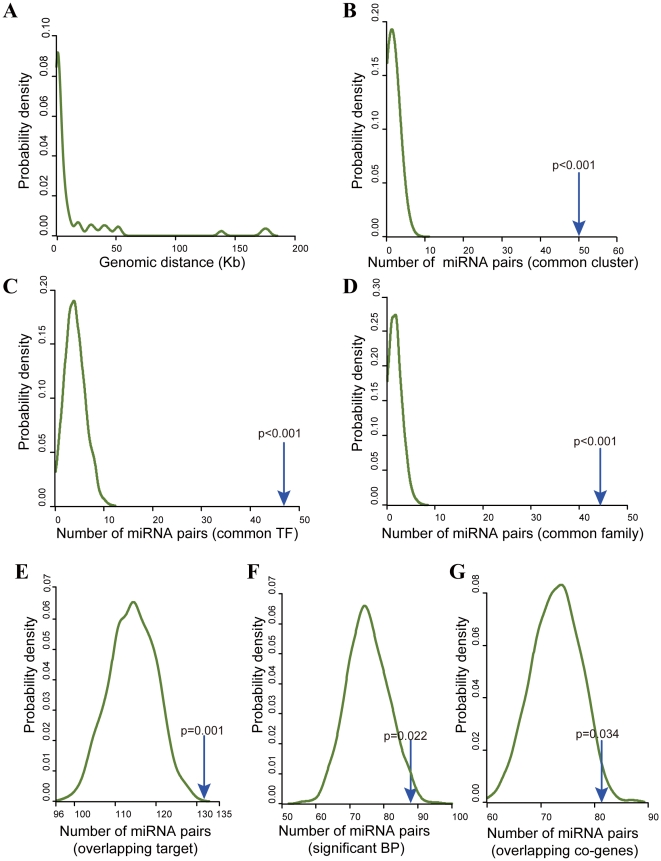
Functional relationships of 182 conserved co-expressed miRNA pairs. (A) Genomic distances of the observed miRNA pairs, which are significantly shorter than the distances of non co-expressed miRNA pairs (Wilcoxon rank sum test, *p-value*<2.2e-16). (B) Probability density of the number of miRNA pairs that belong to the same cluster from randomly selected miRNA pairs. The count observed in the real co-expressed miRNA pairs (50, located by the blue arrow) is significantly higher than those in the random pairs (*p-value*<0.001). (C) Probability density of the number of miRNA pairs that share common TFs from randomly selected miRNA pairs. The count observed in the real co-expressed miRNA pairs (47, located by the blue arrow) is significantly higher than those in the random pairs (*p-value*<0.001). (D) Probability density of the number of miRNA pairs belonging to the same family from randomly selected miRNA pairs. The count observed in the real co-expressed miRNA pairs (44, located by the blue arrow) is significantly higher than those in the random pairs (*p-value*<0.001). (E) The number of miRNA pairs with significantly overlapping targets in the real conserved co-expression pairs (132, located by the blue arrow) is significantly higher than those in the randomly selected miRNA pairs (*p-value* = 0.001). (F) The number of miRNA pairs with common targets significantly involved in at least one biological process in the real conserved co-expression pairs (88, located by the blue arrow) is significantly higher than those in the randomly selected miRNA pairs (*p-value* = 0.022). (G) The number of miRNA pairs with significantly overlapping expression-related genes in the real conserved co-expression pairs (81, located by the blue arrow) is significantly higher than those in the randomly selected miRNA pairs (*p-value* = 0.034).

Firstly, we examined genomic distances between conserved co-expressed miRNAs. Genomic distances of 64 pairs of miRNAs located on the same chromosome were computed. We found 32 pairs of miRNAs (50%) within 1 Kb of each other, 58 (90.6%) within 50 Kb (Figure 2A). These conserved co-expressed miRNA pairs are significantly closer to each other than non co-expressed miRNA pairs (Wilcoxon rank sum test, *p-value*<2.2e-16). Similarly, 50 pairs of miRNAs belonging to the same miRNA cluster were observed. We found that the real conserved co-expressed miRNA pairs have significantly more pairs of miRNAs within the same cluster than randomly selected miRNA pairs (*p-value*<0.001, Figure 2B). These results, consistent with those reported earlier by Baskerville et al. [25], suggest that these co-expressed miRNAs within a short distance may derive from common transcriptions.

Regulation of miRNA gene transcription is similar to that of protein-coding gene transcription, which is controlled by many TFs. In order to investigate whether these co-expressed miRNA pairs are regulated by common TFs, we obtained 260 experimentally validated TF-miRNA regulation relationships from the TransmiR database [26]. We observed that 47 pairs of miRNAs are co-regulated by at least one common TF, significantly more than randomly selected miRNA pairs (*p-value*<0.001, Figure 2C). This reflects that the co-expression of miRNAs may be resulted from common regulation, indicating their potential functional relevance.

Emerging evidence shows that members of a miRNA family (such as the let-7 family) have similar functions since an abundance of overlapping targets resulted from their common seed sequences. We observed 44 pairs of miRNAs belonging to the same family, significantly more than randomly selected pairs of miRNAs (*p-value*<0.001, Figure 2D).

To validate whether co-expressed miRNAs have functional associations, we obtained their predicted targets from the TargetScan5.1 database [27]. Considering a large number of targets predicted by TargetScan5.1, we used a ‘context score’ (−0.3), which corresponds to a certain magnitude of regression, as a cutoff to screen targets regardless of conservation status of binding sites. Among 182 pairs of miRNAs, 149 pairs in which both members have targets were analyzed. We found that 132 pairs of miRNAs have significantly more overlapping targets than expected by chance (hypergeometric distribution, FDR-corrected *p-value*<0.05). When compared with randomly selected miRNA pairs, more pairs of miRNAs having significant overlapping targets were identified in these real conserved co-expressed miRNA pairs (*p-value* = 0.001, Figure 2E). Furthermore, functional enrichment analyses identified 88 miRNA pairs with common targets significantly involved in at least one biological process, significantly more than randomly selected pairs of miRNAs (*p-value* = 0.022, Figure 2F).

Subsequently, we obtained an additional paired miRNA and mRNA expression dataset (GSE25692), and then calculated Pearson correlation coefficients (PCCs) between miRNAs and genes. For each miRNA, genes with absolute PCC>0.5 were regarded as its expression-related genes. Using the hypergeometric distribution, we identified 81 miRNA pairs with significantly overlapping expression-related genes, significantly more than randomly selected pairs of miRNAs (*p-value* = 0.034, Figure 2G).

Moreover, we sought to use miRNA-affected genes generated by knockout/transfection of miRNAs to further determine function associations of these conserved co-expressed miRNA pairs. Multiple gene expression data sets referring to knockout/transfection of different miRNAs were obtained from Gene Expression Omnibus (GEO). Sixteen miRNA pairs in which both members have knockout/transfection data were used. Genes with FC>1.2 were regarded as miRNA-affected genes. Using the hypergeometric distribution, we found that these pairs have significantly more overlapping affected genes except one (Table S2).

Together, our findings suggest strong functional relationships between conserved co-expressed miRNAs, which may be derived from common clusters and/or common families, and can be regulated by common TFs. More importantly, miRNAs can cooperatively regulate a single biological process by targeting common components of that process, which may be the crucial mechanism underlying the ‘fine-tuning’ of gene expression.

### Dysfunction of miRNA cooperation in disease

**Figure 3:**
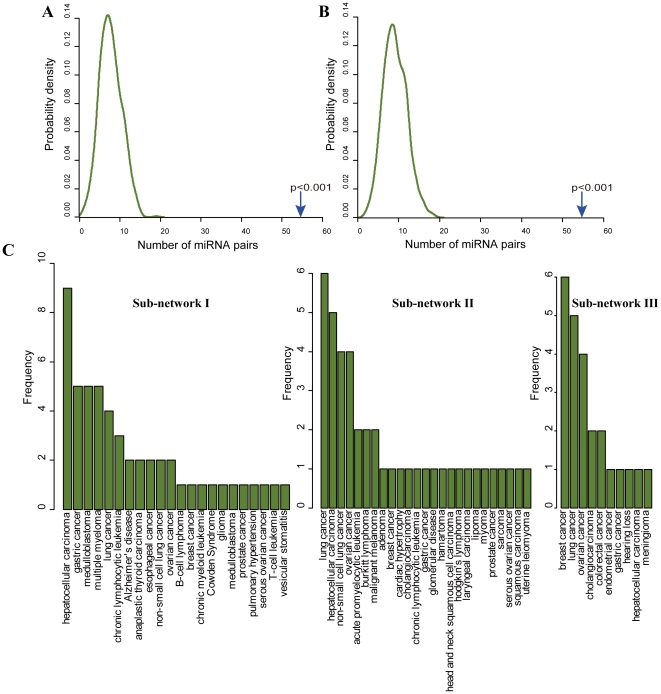
Dysfunction of multiple co-expressed microRNAs in a common disease. (A) The distribution of the number of miRNA pairs sharing a common disease from random selections of miRNA pairs. The number observed in the real conserved co-expression pairs (located by the blue arrow) is significantly higher than those in the randomly selected miRNA pairs (*p-value*<0.001). (B) The distribution of the number of miRNA pairs sharing a common disease from random selections of disease miRNAs. The number observed in the real conserved co-expression pairs (located by the blue arrow) is significantly higher than those from the randomly selected disease miRNAs (*p-value*<0.001). (C) The numbers of miRNAs associated with different human diseases in each sub-network.

Currently, many miRNAs have been identified as disease miRNAs, which play important roles in the development and progression of various diseases. Is the functional cooperation between miRNAs associated with disease? We obtained disease miRNAs from the miR2Disease database [28], and then 204 miRNAs with ‘causal’ relations were gained. Among them, 73 disease miRNAs can be mapped to the network. We identified 54 miRNA pairs sharing a common disease. We verified the significance by means of permuting co-expressed miRNA pairs and disease miRNAs separately. First, we generated 1000 random sets, each of which is composed of 182 randomly selected miRNA pairs. We found that the real conserved co-expressed miRNA pairs have significantly more pairs sharing a common disease than randomly selected miRNA pairs (*p-value*<0.001, Figure 3A). We next evaluated the significance by constructing 1000 sets of randomly selected disease miRNAs. We compared the observed number with the distribution of the number of miRNA pairs sharing a common disease seen in the 1000 randomly generated disease miRNA sets. The similar result was observed (*p-value*<0.001, Figure 3B). These results indicate that dysfunction of function-related miRNAs may be associated with the pathogenesis of disease.

**Figure 4:**
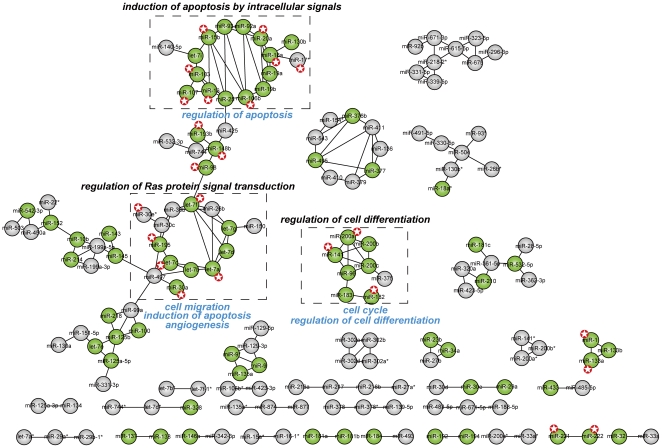
The human conserved miRNA co-expression network. Known disease miRNAs (green) were mapped onto the network. Three disease-related miRNA sub-networks in the dashed boxes were identified. MiRNAs with knockout/transfection experiments are labeled with red stars. Biological processes significantly overrepresented in common targets of each sub-network are recorded on top of the corresponding dashed box with black color. Using miRNAs with knockout/transfection experiments in each sub-network, biological processes significantly overrepresented in consistently affected genes are recorded at the bottom of the corresponding dashed box with blue color.

Subsequently, we identified several highly connected sub-networks using random walks [29]. We observed that some sub-networks are obviously enriched by disease miRNAs (Figure 4). The three sub-networks containing the most disease miRNAs were identified. The sub-network I, II and III contain 16, 14 and 8 miRNAs, respectively. To our surprise, we found that multiple miRNAs in each sub-network are significantly involved in the same diseases (Figure 3C). For example, 14 miRNAs in the sub-network I are disease miRNAs. Of these, 9 miRNAs have been identified as important factors contributing to the development of hepatocellular carcinoma. Furthermore, the remaining 5 disease miRNAs in this sub-network are also associated with various other cancers. In the sub-network III, 6 of 8 miRNAs are breast cancer-related miRNAs. These findings indicate that carcinogenesis may be associated with dysfunction of multiple function-related miRNAs in these sub-networks rather than single miRNAs.

To determine whether these miRNA sub-networks are involved in cancer-related biological processes, we obtained their predicted targets using TargetScan5.1 (without the ‘context score’ limitation). In the sub-network I and II, 64 and 90 common targets shared by at least 13 and 12 miRNAs were identified, respectively. In the sub-network III, 54 common targets regulated by all 8 miRNAs were identified. Through functional enrichment analyses of these sets of common targets, we found that the sub-network I, II and III is significantly involved in ‘induction of apoptosis by intracellular signals’, ‘regulation of Ras protein signal transduction’ and ‘regulation of cell differentiation’ respectively, suggesting that miRNAs in each sub-network can cooperatively regulate important cancer-related biological processes by targeting common transcripts.

Especially, many miRNAs in the sub-networks are from the same family. This might make the prediction of their functions based on their targets biased, because miRNAs in the same family have similar targets. Therefore, we re-predicted the functions of these sub-networks by eliminating the affection of miRNAs from the same family. In each sub-network, common targets of miRNAs from the same family were obtained, and then these family-based target sets together with target sets of other miRNAs not belonging to the same family were used to determine the functions of the sub-network. The sub-network I contains a total of 16 miRNAs, with 15 having predicted targets. In the sub-network I, 12 miRNAs are from 5 miRNA families. For each miRNA family, their common targets were determined. Then, the 5 family-based target sets and 3 target sets from the remaining 3 miRNAs were obtained. We identified 158 common targets shared by at least 6 of these 8 target sets. By function enrichment, we found ‘induction of apoptosis by intracellular signals’ significantly overrepresented in the common targets. Similarly, we identified ‘regulation of Ras protein signal transduction’ and ‘regulation of cell differentiation’ significantly overrepresented in sub-network II and III, respectively. These results are obviously consistent with the above results determined using all predicted targets.

In addition, we determined the functions of these sub-networks based on a paired miRNA and mRNA expression data set (GSE25692). For each miRNA, genes with absolute PCC>0.5 were identified as its expression-related genes. In the sub-network I, 13 miRNAs have expression values in the miRNA expression dataset. 64 common expression-related genes shared by at least 11 miRNAs were identified. We found that these common genes are significantly involved in ‘negative regulation of NF-kappaB import into nucleus’, which plays an important role in apoptosis. Likewise, we found that the sub-network II and III are significantly involved in ‘regulation of Rab GTPase activity’ and ‘cell cycle’, respectively.

To further validation the functions of the three sub-networks, we identified consistently affected genes for each sub-network based on expression data sets from different miRNA knockout/transfection experiments. In the sub-network I, knockout/transfection expression data of 8 miRNAs were collected and their consistently affected genes (FC>1.2) were identified. The process of ‘regulation of apoptosis’ is significantly overrepresented in their common affected genes. For the sub-network II, three biological processes including ‘induction of apoptosis’, ‘cell migration’ and ‘angiogenesis’ are significantly enriched in their common affected genes in 6 miRNAs. Likewise, based on knockout/transfection expression data of 3 miRNAs in the sub-network III, we found that their common affected genes are significantly involved in ‘regulation of cell differentiation’ and ‘cell cycle’. For the sub-network I and III, these results are obviously consistent with those determined using predicted targets. For the sub-network II, although without any consistent processes, the Ras signaling pathway can control apoptosis and cell migration, and its abnormality can lead to increased invasion and metastasis. Taken together, our findings suggest that these three sub-networks play important roles in cancer, further supporting the dysfunction of cooperation of miRNAs in disease.

Furthermore, we found that these disease-related sub-networks are composed of different clusters and families as well as other unrelated miRNAs. The sub-network I is composed of three clusters including miR-17-92, miR-106b-25 and miR-15b-16 clusters, and two paralogous miRNAs miR-103 and miR-107 as well as three unrelated miRNAs including let-7i, miR-130b and miR-140-5p. Of these 16 miRNAs, 14 are identified as disease miRNAs. We found that this sub-network is associated with hepatocellular carcinoma, and can be cooperatively involved in regulation of apoptosis by analyzing predicted targets and affected genes from knockout/transfection of miRNAs. The miR-17-92 cluster has been widely demonstrated to be involved in cell proliferation, apoptosis and angiogenesis in different types of cancers [30]. The miR-106b-25 cluster, one miR-17-92 paralogous cluster, also has important roles in hepatocellular carcinoma. Li *et al*. [31] revealed consistent aberrant expression of these two paralogous clusters in hepatocellular carcinoma. A recent work completed by Ventura *et al.* [32] showed that the miR-17-92 and miR-106b-25 double knockout mice have more severe phenotype than that of miR-17-92 single knockout mice. For the miR-15b-16 cluster, Guo *et al*. [33] found that its two members miR-15b and miR-16 are essential for apoptosis in the rat hepatic stellate cells. The evidence further confirmed our results that these three miRNA clusters may function together to cooperatively control apoptosis, and their impairments may contribute to hepatocellular carcinoma.

The sub-network II is composed of six members of the let-7 family (let-7a, let-7b, let-7c, let-7d, let-7f, and let-7g), the miR-30 family, the miR-195/miR-497 cluster, and two unrelated miRNAs including miR-26b and miR-150. Of these 14 miRNAs, 8 are disease miRNAs, and 6 are related to lung cancer. Functional enrichment analyses based on targets show that this sub-network is significantly involved in regulation of Ras protein signal transduction. Analysis of affected genes from knockout/transfection of miRNAs also shows similar results. The let-7 family has been widely identified to be a tumor suppressor that can inhibit cell proliferation in lung cancer. A recent study demonstrated that the miR-195/497 cluster can suppress cell proliferation and invasion of breast cancer [34]. For the miR-30 family, its function is unclear. Our results indicate that the miR-30 family may function together with the let-7 family and the miR-195/497 cluster to control cell proliferation by cooperatively destroying the Ras signaling pathway. It is notable that miR-26b is highly connected with four let-7 family members including let-7a, let-7d, let-7f and let-7g. The function of miR-26b is unclear. Using predicted targets, we found that all of these miRNAs in the sub-network can consistently regulate Lin28B, which encodes a highly conserved RNA-binding protein, and has important roles in oncogenesis. Therefore, it is rational to presume that miR-26b may function together with these four let-7 family members to consistently regress the expression of Lin28B, and dysfunction of their functional cooperation may lead to up-regulation of Lin28B, which in turn contribute to tumorigenesis. The sub-network III is composed of the miR-200 family (including miR-200a, miR-200b, miR-200c, and miR-141), the miR-183-96-182 cluster, and miR-375. Of these 8 miRNAs, 7 are disease miRNAs, and 6 are related to breast cancer. Functional enrichment analysis using predicted targets and affected genes from miRNA knockout/transfection data consistently show that the network is significantly involved in the regulation of cell differentiation. A recent study revealed that the miR-200 family and the miR-183-96-182 cluster are consistently down-regulated in breast cancer stem cells [35]. By analyzing the process ‘regulation of cell differentiation’ enriched by their targets and affected genes, we found two common genes including WNT5A and CDK6. WNT5A is a member of the WNT gene family, which can tightly regulate self-renewal in stem cells and maintain the undifferentiated state of stem cells. Activation of WNT signaling has been identified in many cancers [36]. CDK6 overexpressed in several malignancies (including breast cancer, lymphoma and melanoma) has been demonstrated to be involved in leukemic cell differentiation block [37]. It seems that the common down-regulation of the miR-200 family and the miR-183-96-182 cluster results in the activities of WNT signaling and CDK6, which can control breast cancer stem cell differentiation and self-renewal to allow malignant proliferation.

## Discussion

By combining multiple miRNA expression data sets from human and mouse, we constructed a conserved miRNA co-expression networks. We analyzed several characteristics of these conserved co-expressed miRNA pairs, and found that parts of these miRNA pairs are located in neighboring genomic positions (belonging to common miRNA clusters), indicating that they are generally derived from common transcripts. The remaining pairs are not located in the same chromosome, suggesting a more general mechanism for regulating expressions of miRNAs. By investigating TF-miRNA relationships, we found that co-expressed miRNA pairs tend to be regulated by common TFs. Recent studies demonstrated that miRNAs can frequently participate in regulatory networks with TFs [38], [39], [40], [41], [42], and different types of feedback loops between miRNAs and TFs may play important roles in many developmental processes, such as self-renewal of human embryonic stem cells [43], [44]. Hence, we suspected that these conserved co-expressed miRNAs are finely controlled by TFs.

Analyses of predicted targets demonstrated that these co-expressed miRNAs have similar targets and similar functions. We also used an additional paired miRNA and mRNA expression data set to validate the functional associations of these co-expressed miRNA pairs based on expression-related genes. This strategy has been used to predict miRNA functions [45]. In addition, the application of miRNA knockout/transfection approaches provide the efficient way for analyzing miRNA functions. The affected genes derived from knockout/transfection of a specific miRNA can be its direct targets or second targets. We evaluated the function relations between these co-expressed miRNAs using some knockout/transfection data. All of our findings show that these co-expressed miRNA pairs can cooperatively regulate common genes, suggesting their functional relevance. In addition, because these expression data sets are derived from different tissues and cell lines in different disease states, the conserved co-expressed miRNA pairs represent widespread co-expression relationships, suggesting their general function associations and their potential important roles in cell function.

By mapping known disease miRNAs, we found that many disease miRNAs are co-expressed, and identified three disease-related sub-networks. Particularly, many members in each sub-network are associated with the same disease. Based on predicted targets, paired miRNA and mRNA expression data and affected genes derived from knockout/transfection of miRNAs, we further demonstrated that miRNAs in each sub-network can cooperatively control some crucial cancer-related processes, such as induction of apoptosis and cell differentiation, by targeting common components of those processes. These findings strongly suggest that the development of disease may be associated with dysfunction of cooperation of multiple miRNAs rather than individual miRNAs. That is, the abnormality of single miRNA may be insufficient to impair normal cell function. The functional cooperation between miRNAs may hint that single miRNA can ‘fine-tune’ gene expression, while multiple function-related miRNAs can have ‘big impacts’ on gene expression. Notably, miRNAs with cooperative functions are not merely limited in the same miRNA clusters or families, suggesting that cooperative functions are dependent on different miRNA cluster and/or family members.

Recently, several studies identified a number of cancer-related miRNA modules. Volinia et al. [24] identified miRNA cliques by constructing cancer networks using miRNA expression. Bandyopadhyay et al. [46] constructed a cancer-miRNA network based on experimentally validated cancer-miRNA relationships, and then recognized a number of cancer-miRNA modules. Comparing with their results, we found that many cancer-related miRNA modules reported in the previous studies were also identified in our study. For example, three miRNAs (including miR-106b, miR-93 and miR-20a), which were identified as a miRNA module in [24], were included in the sub-network I. Three miRNAs (including let-7a, let-7d and let-7f), which were identified as a miRNA module in [46], were included in the sub-network II. Interestingly, in the cancer network constructed in [24], 8 members of the sub-network II (including miR-30c, miR-30b, let-7a, let-7b, let-7c, let-7d, let-7f and let-7g) were found to be located in two neighboring modules.

Note that there are 44 pairs of co-expressed miRNAs in the same family, which may result from the redundancy or lack of probe specificity in the experiments. To rule out this possibility, we measured expression correlations between these 44 pairs of miRNAs using additional three deep sequencing-based (GSE20592, GSE18012 and GSE15229) and one PCR-based expression data sets (GSE23024). We observed that these 44 miRNA pairs exhibit high expression correlations in these expression datasets (Figure S1), suggesting that the co-expressions of miRNAs belonging to the same family are biologically meaningful, not due to the non-specificity of probes in the experiments. Also, we found some members of miRNA families having distinct expression patterns. For example, three miRNAs including miR-25, miR-92a and miR-92b are from the miR-25 family. MiR-25 and miR-92a show a high expression correlation (PCC = 0.798), whereas miR-92b has distinct expression patterns with miR-25 and miR-92a. The expression correlation between miR-92a and miR-92b is 0.2, and the expression correlation between miR-25 and miR-92b is 0.172. Both miR-25 and miR-92a were found to play roles in cell proliferation [31], [47]. MiR-92b was found to suppress pro-inflammatory responses [48]. This indicates that not all miRNAs with similar seeds can perform similar functions.

In particular, because many co-expressed miRNA pairs are belonging to the same family and likely have the same or similar seeds, this might make our results (i.e., these co-expressed miRNAs tend to be proximally located in the genome, belong to the same clusters, share the same TFs, target similar genes, and have similar functions) biased. Hence, we re-evaluated the 138 co-expressed miRNA pairs not belonging to the same family. We found that these 138 co-expressed miRNA pairs show the similar tendency as observed in the whole 182 co-expressed miRNA pairs (Figure S2).

It should be noted that using knockout/transfection data was a more effective method for analyzing the functional relations between co-expressed miRNAs, although targets and expression-related genes were used to comprehensively validate the functional relations. However, there were only few knockout/transfection data available. We only analyzed 16 pairs of co-expressed miRNAs using knockout/transfection data. Unfortunately, many miRNA pairs validated using knockout/transfection data were between family members. Thus, more knockout/transfection data should be used to further demonstrate the functional relations between co-expressed miRNAs.

In summary, we constructed a conserved miRNA co-expression network by combining human and mouse miRNA expression data sets. We demonstrated close functional relationships between these conserved co-expressed pairs of miRNAs. Moreover, we identified three disease-related sub-networks and found that members in each sub-network can cooperatively regulate some cancer-related biological processes (e.g. regulation of apoptosis). Our results hint that human disease may be associated with the impairment of functional cooperation of multiple miRNAs rather than single miRNAs. Therefore, understanding miRNA function only dependent on single miRNAs is difficult. Instead, analyzing functionally cooperative miRNAs may be an important step for deciphering the complex function of miRNAs and their roles in human disease.

## Materials and Methods

### MiRNA expression data sets

We collected 16 human and 8 mouse miRNA expression data sets profiling using Agilent arrays from GEO (human: GSE14985, GSE15144, GSE16444, GSE17498, GSE18469, GSE18470, GSE18999, GSE19232, GSE19347, GSE19544, GSE19783, GSE21036, GSE21687, GSE23690, GSE24485 and GSE25508; mouse: GSE14267, GSE17000, GSE18786, GSE19421, GSE19487, GSE21003, GSE21798 and GSE24321). All miRNA expression data sets were normalized by using the quantiles method, as implemented in the Bioconductor affy package [49]. Since miRNA probes from various Agilent platforms were designed based on different miRBase versions, probe sequences were mapped to known mature miRNA sequences from the miRBase [50] database (version 16.0). Probes mapped to multiple miRNAs were removed. In cases where a single miRNA is mapped by multiple probes, the median value was used. For each species, miRNAs present in all microarray platforms were used for the combination of expression data sets.

### Identification of orthologous miRNAs

A typical animal primary miRNA (pri-miRNA) contains a hairpin stem of 33 bp, a terminal loop, and two single-stranded flanking segments. Drosha and DGCR8 formed ‘Microprocessor’ play essential roles in pri-miRNA processing, and their processing center is placed at the double-stranded stem, ∼11 bp from the junction between the stem and flanking regions [51]. Because the sequences of most pri-miRNAs are unknown, we obtained human and mouse pre-miRNA sequences including their flanking sequences (upstream and downstream 11 bp) using the UCSC database [52]. Orthologs were identified by performing all-against-all alignment between every pair of sequences from human and mouse using the BLAT algorithm. Only the miRNA pairs that show best reciprocal hits and have identical seed regions (nucleotides 2–7) were regarded as orthologous miRNAs.

### Construction of a conserved miRNA co-expression network

In order to identity conserved co-expressed miRNAs across human and mouse, a previously proposed method by Stuart *et al.* [20] was applied. Given a species, we calculated pairwise Pearson correlation coefficients for all miRNAs in the species., For each miRNA, we ranked all of the other miRNAs relative to this miRNA based on their correlation coefficients in a descending order and then calculated rank ratios by dividing the ranks by the total number of miRNAs in the species. For a miRNA pair A–B, the rank ratio of miRNA A relative to B and the rank ratio of miRNA B relative to A were recorded. The minimum rank ratio was used to represent the correlation coefficient of this miRNA pair. Likewise, the rank ratio for its ortholog miRNA pair A′–B′ in the other species was computed as above. Finally, for each miRNA pair in human and its corresponding ortholog pair in mouse, two rank ratios were obtained. Based on the technique of order statistics, the probability of getting the observed rank ratios by chance was calculated using the joint cumulative distribution of an *n*-dimensional order statistics.
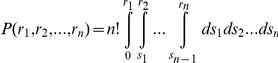



The above integral was computed using the following recursive formula:
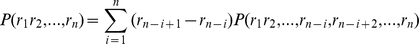
where *r_i_* is the rank ratio for species *i*, *n* is the number of species (*n* = 2 in this analysis), and *r*_0_ = 0. We then performed multiple testing correction using the Bonferroni method, with a *p-value*<0.05 considered as statistically significant.

### MiRNA genomic location, cluster, family, TF, targets and disease information

MiRNA genomic location, cluster (defined by a 10 Kb threshold) and family information were retrieved from the miRBase database (version 16.0). Experimentally validated TF-miRNA regulation relationships were gained from the TransmiR database [26]. Targets of miRNAs were predicted using TargetScan 5.1 [27]. All known disease miRNAs with ‘causal’ relationships were collected from the miR2Disease database [28].

### Gene expression data from miRNA knockout/transfection experiments

Gene expression data sets referring to knockout/transfection of single miRNAs were collected from the GEO (GSE2075, GSE6474, GSE6838, GSE8501, GSE11968, GSE12615, GSE14847, GSE19688, GSE19777 and GSE22002). In each miRNA knockout/transfection experiment, fold changes of all genes were calculated by comparing expression values between cases and controls. Genes with fold change (FC)>1.2 were identified as differential genes. If a single miRNA is knockout or overexpressed in multiple different cell lines, the union of the differential genes in all cell lines was defined as the miRNA-affected gene set.

### Functional enrichment analysis

For a set of genes, overrepresented biological processes were determined using the hypergeometric distribution based on Gene Ontology (GO). *P-value* of 0.001 was used as the cutoff value to identify significantly overrepresented biological processes. Functional enrichment analysis was performed using the Bioconductor package GOstats [53].

## Supporting Information

Figure S1 Probability density of the Pearson correlation coefficients of the 44 co-expressed miRNA pairs belonging to the same family. The Pearson correlation coefficients were calculated using three deep sequencing- and one PCR-based expression data sets.
(TIF)

Figure S2 Functional relationships of 138 conserved co-expressed miRNA pairs not belonging to the same family. (A) Genomic distances of the observed miRNA pairs, which are significantly lower than the distances of non co-expressed miRNA pairs (Wilcoxon rank sum test, *p-value*<4.963e-14). (B) Distributions Probability density of the number of miRNA pairs that belong to the same cluster from randomly selected miRNA pairs. The count observed in the real co-expressed miRNA pairs (25, located by the blue arrow) is significantly higher than those in the random pairs (*p-value*<0.001). (C) Probability density of the number of miRNA pairs that share common TFs from randomly selected miRNA pairs. The count observed in the real co-expressed miRNA pairs (26, located by the blue arrow) is significantly higher than those in the random pairs (*p-value*<0.001). (D) The number of miRNA pairs with significantly overlapping targets in the real conserved co-expression pairs (99, located by the blue arrow) is significantly higher than those in the randomly selected miRNA pairs (*p-value* = 0.02).
(TIF)

Table S1 253 human-mouse mature orthologous miRNAs.
(DOC)

Table S2 Significance of overlapping affected genes between conserved co-expressed miRNA pairs using miRNA knockout/transfection expression data.
(DOC)
