# Supplementary figures and images for: Discovering Dysfunction of Multiple MicroRNAs Cooperation in Disease by a Conserved MicroRNA Co-Expression Network
Source: PLoS One. 2012 Feb 22;7(2):e32201. doi: 10.1371/journal.pone.0032201 (PMC3285207; doi:10.1371/journal.pone.0032201)

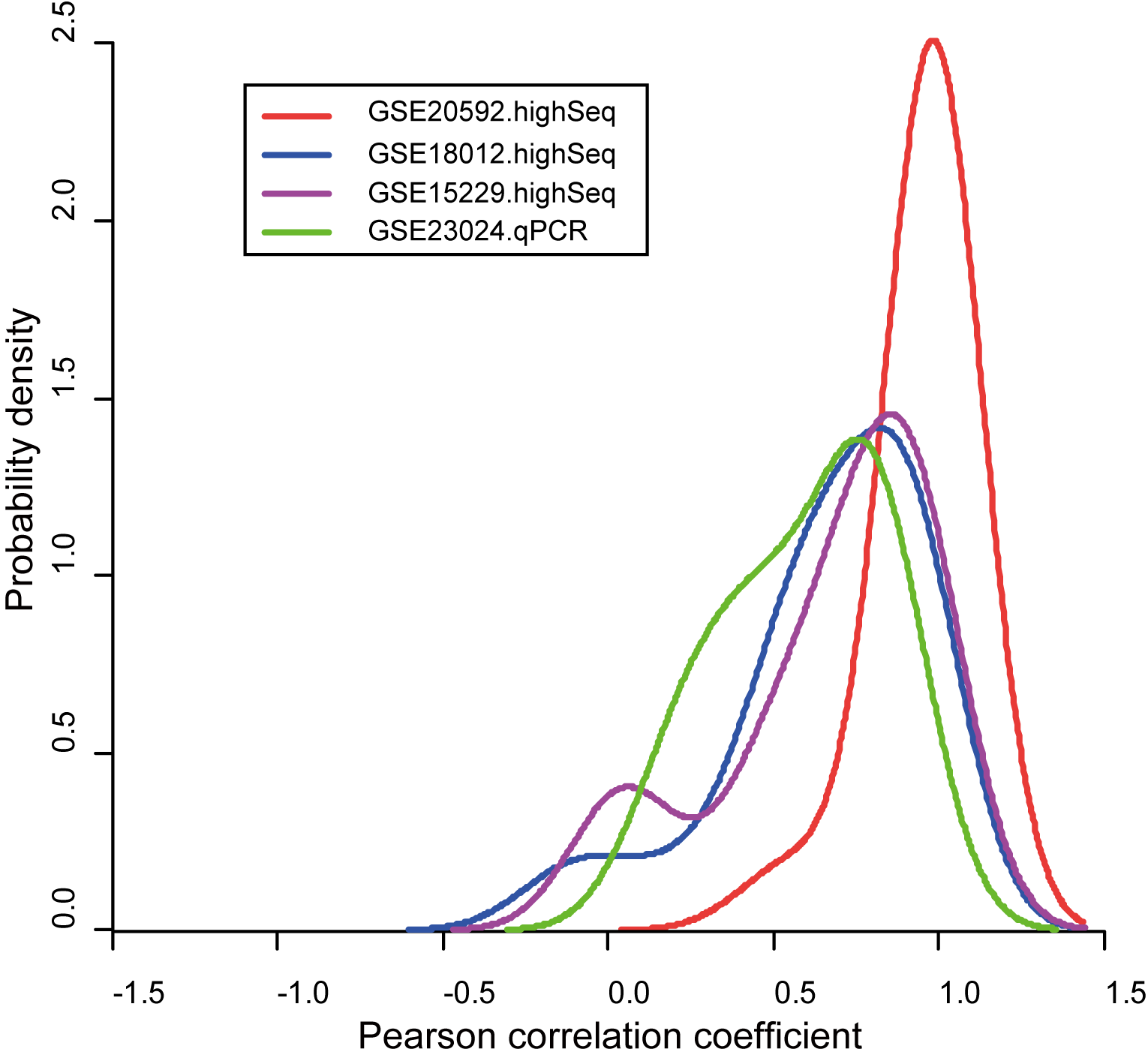

Supplement: Figure S1 — Probability density of the Pearson correlation coefficients of the 44 co-expressed miRNA pairs belonging to the same family. The Pearson correlation coefficients were calculated using three deep sequencing- and one PCR-based expression data sets. (TIF) [file pone.0032201.s001.tif]

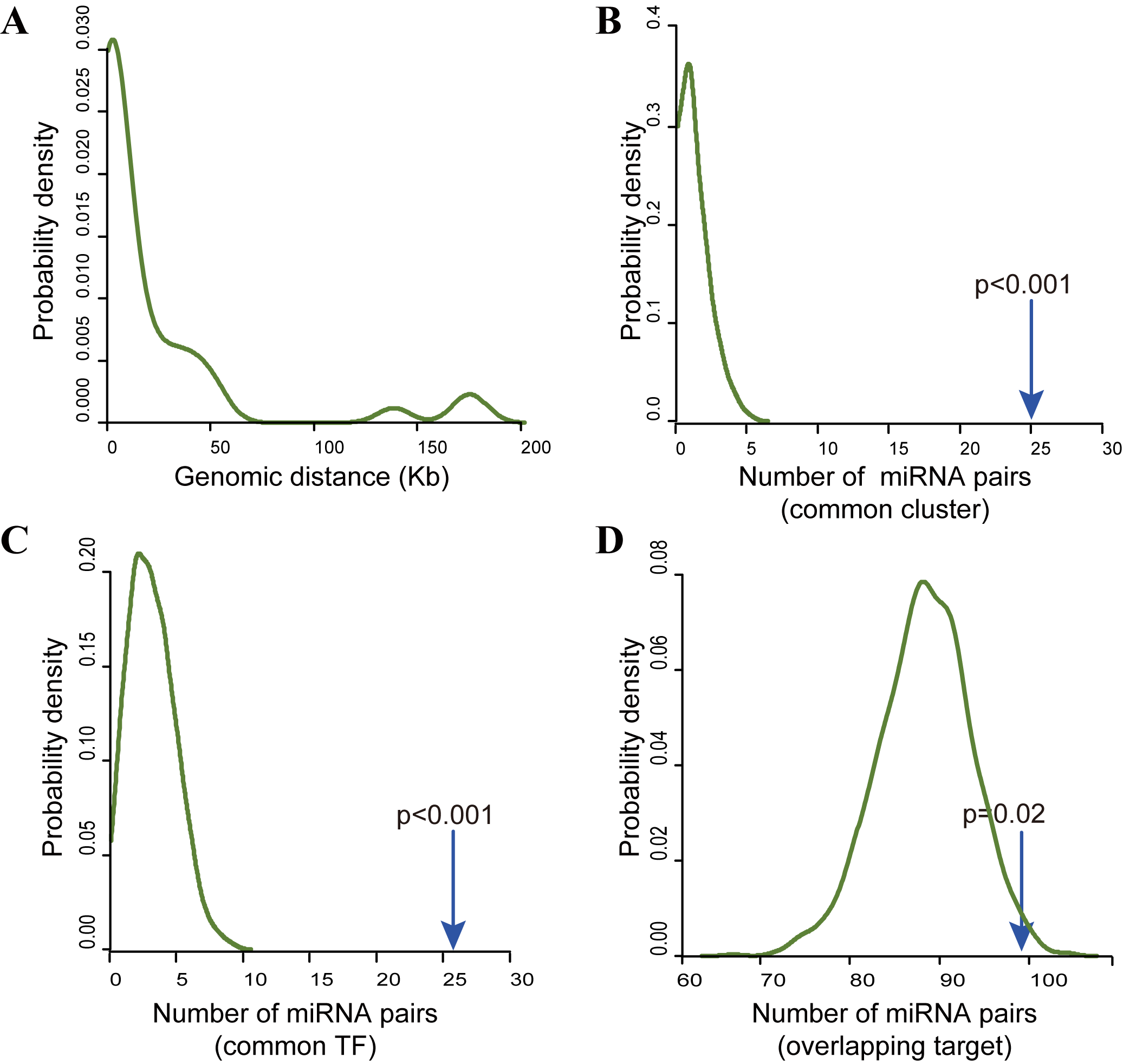

Supplement: Figure S2 — Functional relationships of 138 conserved co-expressed miRNA pairs not belonging to the same family. (A) Genomic distances of the observed miRNA pairs, which are significantly lower than the distances of non co-expressed miRNA pairs (Wilcoxon rank sum test, p-value<4.963e-14). (B) Distributions Probability density of the number of miRNA pairs that belong to the same cluster from randomly selected miRNA pairs. The count observed in the real co-expressed miRNA pairs (25, located by the blue arrow) is significantly higher than those in the random pairs (p-value<0.001). (C) Probability density of the number of miRNA pairs that share common TFs from randomly selected miRNA pairs. The count observed in the real co-expressed miRNA pairs (26, located by the blue arrow) is significantly higher than those in the random pairs (p-value<0.001). (D) The number of miRNA pairs with significantly overlapping targets in the real conserved co-expression pairs (99, located by the blue arrow) is significantly higher than those in the randomly selected miRNA pairs (p-value = 0.02). (TIF) [file pone.0032201.s002.tif]
