# Supplementary material for: Discovering Dysfunction of Multiple MicroRNAs Cooperation in Disease by a Conserved MicroRNA Co-Expression Network
Source: PLoS One. 2012 Feb 22;7(2):e32201. doi: 10.1371/journal.pone.0032201 (PMC3285207; doi:10.1371/journal.pone.0032201)
Supplement: Table S1 — 253 human-mouse mature orthologous miRNAs. (DOC) [file pone.0032201.s003.doc]

Supplemental Table S1. 253 human-mouse mature orthologous miRNAs

| **Human** | **Mouse** |
| --- | --- |
| hsa-let-7a | mmu-let-7a |
| hsa-let-7a* | mmu-let-7a-1* |
| hsa-let-7b | mmu-let-7b |
| hsa-let-7b* | mmu-let-7b* |
| hsa-let-7c | mmu-let-7c |
| hsa-let-7d | mmu-let-7d |
| hsa-let-7d* | mmu-let-7d* |
| hsa-let-7e | mmu-let-7e |
| hsa-let-7f | mmu-let-7f |
| hsa-let-7f-1* | mmu-let-7f-1* |
| hsa-let-7g | mmu-let-7g |
| hsa-let-7i | mmu-let-7i |
| hsa-let-7i* | mmu-let-7i* |
| hsa-miR-1 | mmu-miR-1 |
| hsa-miR-100 | mmu-miR-100 |
| hsa-miR-101 | mmu-miR-101a;mmu-miR-101b |
| hsa-miR-103 | mmu-miR-103 |
| hsa-miR-106b | mmu-miR-106b |
| hsa-miR-106b* | mmu-miR-106b* |
| hsa-miR-107 | mmu-miR-107 |
| hsa-miR-10a | mmu-miR-10a |
| hsa-miR-10a* | mmu-miR-10a* |
| hsa-miR-10b | mmu-miR-10b |
| hsa-miR-122 | mmu-miR-122 |
| hsa-miR-125a-3p | mmu-miR-125a-3p |
| hsa-miR-125a-5p | mmu-miR-125a-5p |
| hsa-miR-125b | mmu-miR-125b-5p |
| hsa-miR-129-3p | mmu-miR-129-2-3p |
| hsa-miR-129-5p | mmu-miR-129-5p |
| hsa-miR-130a | mmu-miR-130a |
| hsa-miR-130b | mmu-miR-130b |
| hsa-miR-130b* | mmu-miR-130b* |
| hsa-miR-132 | mmu-miR-132 |
| hsa-miR-133a | mmu-miR-133a |
| hsa-miR-133b | mmu-miR-133b |
| hsa-miR-134 | mmu-miR-134 |
| hsa-miR-135a | mmu-miR-135a |
| hsa-miR-135a* | mmu-miR-135a-1* |
| hsa-miR-136 | mmu-miR-136 |
| hsa-miR-137 | mmu-miR-137 |
| hsa-miR-138 | mmu-miR-138 |
| hsa-miR-139-5p | mmu-miR-139-5p |
| hsa-miR-140-5p | mmu-miR-140 |
| hsa-miR-141 | mmu-miR-141 |
| hsa-miR-141* | mmu-miR-141* |
| hsa-miR-142-3p | mmu-miR-142-3p |
| hsa-miR-143 | mmu-miR-143 |
| hsa-miR-145 | mmu-miR-145 |
| hsa-miR-146a | mmu-miR-146a |
| hsa-miR-146b-5p | mmu-miR-146b |
| hsa-miR-147b | mmu-miR-147 |
| hsa-miR-148a | mmu-miR-148a |
| hsa-miR-148a* | mmu-miR-148a* |
| hsa-miR-148b | mmu-miR-148b |
| hsa-miR-149 | mmu-miR-149 |
| hsa-miR-150 | mmu-miR-150 |
| hsa-miR-150* | mmu-miR-150* |
| hsa-miR-151-3p | mmu-miR-151-3p |
| hsa-miR-151-5p | mmu-miR-151-5p |
| hsa-miR-152 | mmu-miR-152 |
| hsa-miR-153 | mmu-miR-153 |
| hsa-miR-154 | mmu-miR-154 |
| hsa-miR-154* | mmu-miR-154* |
| hsa-miR-15a* | mmu-miR-15a* |
| hsa-miR-15b | mmu-miR-15b |
| hsa-miR-15b* | mmu-miR-15b* |
| hsa-miR-16 | mmu-miR-16 |
| hsa-miR-16-1* | mmu-miR-16-1* |
| hsa-miR-17* | mmu-miR-17* |
| hsa-miR-181a | mmu-miR-181a |
| hsa-miR-181a* | mmu-miR-181a-1* |
| hsa-miR-181b | mmu-miR-181b |
| hsa-miR-181c | mmu-miR-181c |
| hsa-miR-181d | mmu-miR-181d |
| hsa-miR-182 | mmu-miR-182 |
| hsa-miR-183 | mmu-miR-183 |
| hsa-miR-183* | mmu-miR-183* |
| hsa-miR-184 | mmu-miR-184 |
| hsa-miR-185 | mmu-miR-185 |
| hsa-miR-187 | mmu-miR-187 |
| hsa-miR-188-3p | mmu-miR-188-3p |
| hsa-miR-188-5p | mmu-miR-188-5p |
| hsa-miR-18a | mmu-miR-18a |
| hsa-miR-18a* | mmu-miR-18a* |
| hsa-miR-18b | mmu-miR-18b |
| hsa-miR-190 | mmu-miR-190 |
| hsa-miR-190b | mmu-miR-190b |
| hsa-miR-191 | mmu-miR-191 |
| hsa-miR-192 | mmu-miR-192 |
| hsa-miR-193b | mmu-miR-193b |
| hsa-miR-194 | mmu-miR-194 |
| hsa-miR-195 | mmu-miR-195 |
| hsa-miR-196a | mmu-miR-196a |
| hsa-miR-196b | mmu-miR-196b |
| hsa-miR-199a-3p | mmu-miR-199a-3p |
| hsa-miR-199a-5p | mmu-miR-199a-5p |
| hsa-miR-19a | mmu-miR-19a |
| hsa-miR-19b | mmu-miR-19b |
| hsa-miR-200a | mmu-miR-200a |
| hsa-miR-200a* | mmu-miR-200a* |
| hsa-miR-200b | mmu-miR-200b |
| hsa-miR-200b* | mmu-miR-200b* |
| hsa-miR-200c | mmu-miR-200c |
| hsa-miR-200c* | mmu-miR-200c* |
| hsa-miR-203 | mmu-miR-203 |
| hsa-miR-204 | mmu-miR-204 |
| hsa-miR-205 | mmu-miR-205 |
| hsa-miR-20a | mmu-miR-20a |
| hsa-miR-20a* | mmu-miR-20a* |
| hsa-miR-21 | mmu-miR-21 |
| hsa-miR-210 | mmu-miR-210 |
| hsa-miR-214 | mmu-miR-214 |
| hsa-miR-214* | mmu-miR-214* |
| hsa-miR-216a | mmu-miR-216a |
| hsa-miR-216b | mmu-miR-216b |
| hsa-miR-217 | mmu-miR-217 |
| hsa-miR-218 | mmu-miR-218 |
| hsa-miR-218-2* | mmu-miR-218-2* |
| hsa-miR-219-5p | mmu-miR-219-5p |
| hsa-miR-22 | mmu-miR-22 |
| hsa-miR-22* | mmu-miR-22* |
| hsa-miR-221 | mmu-miR-221 |
| hsa-miR-222 | mmu-miR-222 |
| hsa-miR-223 | mmu-miR-223 |
| hsa-miR-224 | mmu-miR-224 |
| hsa-miR-23b | mmu-miR-23b |
| hsa-miR-24 | mmu-miR-24 |
| hsa-miR-25 | mmu-miR-25 |
| hsa-miR-26b | mmu-miR-26b |
| hsa-miR-26b* | mmu-miR-26b* |
| hsa-miR-27a* | mmu-miR-27a* |
| hsa-miR-27b | mmu-miR-27b |
| hsa-miR-27b* | mmu-miR-27b* |
| hsa-miR-28-5p | mmu-miR-28 |
| hsa-miR-296-3p | mmu-miR-296-3p |
| hsa-miR-296-5p | mmu-miR-296-5p |
| hsa-miR-29a | mmu-miR-29a |
| hsa-miR-29a* | mmu-miR-29a* |
| hsa-miR-29b | mmu-miR-29b |
| hsa-miR-29b-1* | mmu-miR-29b-1* |
| hsa-miR-301a | mmu-miR-301a |
| hsa-miR-301b | mmu-miR-301b |
| hsa-miR-302a | mmu-miR-302a |
| hsa-miR-302a* | mmu-miR-302a* |
| hsa-miR-302b | mmu-miR-302b |
| hsa-miR-302b* | mmu-miR-302b* |
| hsa-miR-302d | mmu-miR-302d |
| hsa-miR-30a | mmu-miR-30a |
| hsa-miR-30b | mmu-miR-30b |
| hsa-miR-30c | mmu-miR-30c |
| hsa-miR-30c-1* | mmu-miR-30c-1* |
| hsa-miR-30c-2* | mmu-miR-30c-2* |
| hsa-miR-30d | mmu-miR-30d |
| hsa-miR-30e | mmu-miR-30e |
| hsa-miR-30e* | mmu-miR-30e* |
| hsa-miR-31 | mmu-miR-31 |
| hsa-miR-32 | mmu-miR-32 |
| hsa-miR-320a | mmu-miR-320 |
| hsa-miR-323-5p | mmu-miR-323-5p |
| hsa-miR-324-5p | mmu-miR-324-5p |
| hsa-miR-326 | mmu-miR-326 |
| hsa-miR-328 | mmu-miR-328 |
| hsa-miR-330-5p | mmu-miR-330 |
| hsa-miR-331-3p | mmu-miR-331-3p |
| hsa-miR-331-5p | mmu-miR-331-5p |
| hsa-miR-335 | mmu-miR-335-5p |
| hsa-miR-338-3p | mmu-miR-338-3p |
| hsa-miR-338-5p | mmu-miR-338-5p |
| hsa-miR-339-3p | mmu-miR-339-3p |
| hsa-miR-339-5p | mmu-miR-339-5p |
| hsa-miR-33a | mmu-miR-33 |
| hsa-miR-33a* | mmu-miR-33* |
| hsa-miR-342-3p | mmu-miR-342-3p |
| hsa-miR-342-5p | mmu-miR-342-5p |
| hsa-miR-346 | mmu-miR-346 |
| hsa-miR-34a | mmu-miR-34a |
| hsa-miR-34c-5p | mmu-miR-34c |
| hsa-miR-361-5p | mmu-miR-361 |
| hsa-miR-362-3p | mmu-miR-362-3p |
| hsa-miR-362-5p | mmu-miR-362-5p |
| hsa-miR-367 | mmu-miR-367 |
| hsa-miR-369-3p | mmu-miR-369-3p |
| hsa-miR-369-5p | mmu-miR-369-5p |
| hsa-miR-370 | mmu-miR-370 |
| hsa-miR-375 | mmu-miR-375 |
| hsa-miR-376b | mmu-miR-376b |
| hsa-miR-376c | mmu-miR-376c |
| hsa-miR-377 | mmu-miR-377 |
| hsa-miR-378 | mmu-miR-378 |
| hsa-miR-378* | mmu-miR-378* |
| hsa-miR-379 | mmu-miR-379 |
| hsa-miR-381 | mmu-miR-381 |
| hsa-miR-382 | mmu-miR-382 |
| hsa-miR-409-3p | mmu-miR-409-3p |
| hsa-miR-410 | mmu-miR-410 |
| hsa-miR-411 | mmu-miR-411 |
| hsa-miR-421 | mmu-miR-421 |
| hsa-miR-423-3p | mmu-miR-423-3p |
| hsa-miR-423-5p | mmu-miR-423-5p |
| hsa-miR-425 | mmu-miR-425 |
| hsa-miR-425* | mmu-miR-425* |
| hsa-miR-431 | mmu-miR-431 |
| hsa-miR-431* | mmu-miR-431* |
| hsa-miR-433 | mmu-miR-433 |
| hsa-miR-450a | mmu-miR-450a |
| hsa-miR-451 | mmu-miR-451 |
| hsa-miR-483-5p | mmu-miR-483 |
| hsa-miR-484 | mmu-miR-484 |
| hsa-miR-485-5p | mmu-miR-485 |
| hsa-miR-487b | mmu-miR-487b |
| hsa-miR-488* | mmu-miR-488* |
| hsa-miR-490-3p | mmu-miR-490-3p |
| hsa-miR-491-5p | mmu-miR-491 |
| hsa-miR-493 | mmu-miR-493 |
| hsa-miR-495 | mmu-miR-495 |
| hsa-miR-497 | mmu-miR-497 |
| hsa-miR-503 | mmu-miR-503 |
| hsa-miR-504 | mmu-miR-504 |
| hsa-miR-532-3p | mmu-miR-532-3p |
| hsa-miR-532-5p | mmu-miR-532-5p |
| hsa-miR-539 | mmu-miR-539-5p |
| hsa-miR-542-3p | mmu-miR-542-3p |
| hsa-miR-543 | mmu-miR-543 |
| hsa-miR-551b | mmu-miR-551b |
| hsa-miR-574-3p | mmu-miR-574-3p |
| hsa-miR-574-5p | mmu-miR-574-5p |
| hsa-miR-615-3p | mmu-miR-615-3p |
| hsa-miR-615-5p | mmu-miR-615-5p |
| hsa-miR-652 | mmu-miR-652 |
| hsa-miR-653 | mmu-miR-653 |
| hsa-miR-668 | mmu-miR-668 |
| hsa-miR-671-3p | mmu-miR-671-3p |
| hsa-miR-671-5p | mmu-miR-671-5p |
| hsa-miR-675 | mmu-miR-675-5p |
| hsa-miR-7 | mmu-miR-7a |
| hsa-miR-708 | mmu-miR-708 |
| hsa-miR-708* | mmu-miR-708* |
| hsa-miR-744 | mmu-miR-744 |
| hsa-miR-744* | mmu-miR-744* |
| hsa-miR-874 | mmu-miR-874 |
| hsa-miR-876-5p | mmu-miR-876-5p |
| hsa-miR-877 | mmu-miR-877 |
| hsa-miR-9 | mmu-miR-9 |
| hsa-miR-9* | mmu-miR-9* |
| hsa-miR-92a | mmu-miR-92a |
| hsa-miR-92a-2* | mmu-miR-92a-2* |
| hsa-miR-92b | mmu-miR-92b |
| hsa-miR-93 | mmu-miR-93 |
| hsa-miR-93* | mmu-miR-93* |
| hsa-miR-96 | mmu-miR-96 |
| hsa-miR-98 | mmu-miR-98 |
| hsa-miR-99a | mmu-miR-99a |
| hsa-miR-99b* | mmu-miR-99b* |
