# Supplementary material for: Discovering Dysfunction of Multiple MicroRNAs Cooperation in Disease by a Conserved MicroRNA Co-Expression Network
Source: PLoS One. 2012 Feb 22;7(2):e32201. doi: 10.1371/journal.pone.0032201 (PMC3285207; doi:10.1371/journal.pone.0032201)
Supplement: Table S2 — Significance of overlapping affected genes between conserved co-expressed miRNA pairs using miRNA knockout/transfection expression data. (DOC) [file pone.0032201.s004.doc]

Supplemental Table S2. Significance of overlapping affected genes between conserved co-expressed miRNA pairs using miRNA knockout/transfection expression data

| **miRNA 1** | **miRNA 2** | **Cell line 1** | **Cell line 2** | **GEOID 1** | **GEOID 2** | ***p-values*** |
| --- | --- | --- | --- | --- | --- | --- |
| hsa-miR-16 | hsa-miR-103 | colon cancer cell line | colon cancer cell line | GSE6838 | GSE6838 | 0 |
| hsa-miR-16 | hsa-miR-107 | colon cancer cell line | colon cancer cell line | GSE6838 | GSE6838 | 0 |
| hsa-miR-103 | hsa-miR-107 | colon cancer cell line | colon cancer cell line | GSE6838 | GSE6838 | 0 |
| hsa-miR-16 | hsa-miR-15b | colon cancer cell line | colon cancer cell line | GSE6838 | GSE6838 | 0 |
| hsa-miR-15b | hsa-miR-106b | colon cancer cell line | colon cancer cell line | GSE6838 | GSE6838 | 0 |
| hsa-miR-18a | hsa-miR-20a | breast cancer cell line | glioma cell line | GSE14847 | GSE19688 | 9.98e-41 |
| hsa-miR-18a | hsa-miR-17* | breast cancer cell line | colon cancer cell line | GSE14847 | GSE6838 | 1.98e-05 |
| hsa-miR-193b | hsa-miR-148b | breast cancer cell line | HeLa cell line | GSE14847 | GSE8501 | 1.14e-72 |
| hsa-miR-98 | hsa-miR-148b | ovarian cancer cell line | HeLa cell line | GSE12615 | GSE8501 | 0.95 |
| hsa-miR-98 | hsa-let-7f | ovarian cancer cell line | ovarian cancer cell line | GSE12615 | GSE12615 | 2.42e-35 |
| hsa-let-7a | hsa-let-7c | lung cancer cell line | colon cancer cell line | GSE6474 | GSE6838 | 9.25e-57 |
| hsa-let-7a | hsa-let-7f | lung cancer cell line | ovarian cancer cell line | GSE6474 | GSE12615 | 2.77e-130 |
| hsa-let-7c | hsa-miR-195 | colon cancer cell line | colon cancer cell line | GSE6838 | GSE6838 | 4.94e-324 |
| hsa-miR-200a | hsa-miR-141 | colon cancer cell line | colon cancer cell line | GSE6838 | GSE6838 | 0 |
| hsa-miR-1 | hsa-miR-133a | HeLa cell line | HeLa cell line | GSE2075 GSE11968 GSE22002 | GSE8501 | 1.61e-116 |
| hsa-miR-221 | hsa-miR-222 | breast cancer cell line | breast cancer cell line | GSE19777 | GSE19777 | 0 |
